# Subcutaneous Progesterone Is Effective and Safe for Luteal Phase Support in IVF: An Individual Patient Data Meta-Analysis of the Phase III Trials

**DOI:** 10.1371/journal.pone.0151388

**Published:** 2016-03-18

**Authors:** Jakob Doblinger, Barbara Cometti, Silvia Trevisan, Georg Griesinger

**Affiliations:** 1 Department of Obstetrics and Gynecology, Paracelsus Medical University, Salzburg, Austria; 2 IBSA Institut Biochimique SA, R&D Scientific Affairs, Lugano, Switzerland; 3 Department of Gynecological Endocrinology and Reproductive Medicine, University Hospital of Schleswig-Holstein, Campus Luebeck, Luebeck, Germany; University of Crete, GREECE

## Abstract

**Objective:**

To summarize efficacy and safety data on a new progesterone compound which is available for subcutaneous administration as compared to vaginally administered progesterone for luteal phase support in patients undergoing IVF treatment.

**Design:**

Data from two randomized phase III trials (07EU/Prg06 and 07USA/Prg05) performed according to GCP standards with a total sample size of 1435 per-protocol patients were meta-analyzed on an individual patient data level.

**Setting:**

University affiliated reproductive medicine unit.

**Patients:**

Subcutaneous progesterone was administered to a total of 714 subjects and vaginal progesterone was administered to a total of 721 subjects who underwent fresh embryo transfer after ovarian stimulation followed by IVF or ICSI. The subjects were between 18 and 42 years old and had a BMI <30kg/m^2^.

**Interventions:**

Subcutaneous progesterone 25 mg daily vs. either progesterone vaginal gel 90 mg daily (07EU/Prg06) or 100 mg intravaginal twice a day (07USA/Prg05) for luteal phase support in IVF patients.

**Main outcome measures:**

Ongoing pregnancy rate beyond 10 gestational weeks, live birth rate and OHSS risk.

**Results:**

The administration of subcutaneous progesterone versus intra-vaginal progesterone had no impact on ongoing pregnancy likelihood (OR = 0.865, 95% CI 0.694 to 1.077; P = n.s.), live birth likelihood (OR = 0.889, 95% CI 0.714 to 1.106; P = n.s.) or OHSS risk (OR = 0.995, 95% CI 0.565 to 1.754; P = n.s.) in regression analyses accounting for clustering of patients within trials, while adjusting for important confounders. Only female age and number of oocytes retrieved were significant predictors of live birth likelihood and OHSS risk.

**Conclusion:**

No statistical significant or clinical significant differences exist between subcutaneous and vaginal progesterone for luteal phase support.

## Introduction

Luteal phase support with progesterone is a routine part of IVF treatment. A recent Cochrane review demonstrated that luteal phase support with progesterone is associated with higher rates of live birth or ongoing pregnancy as compared to placebo [1]. Progesterone is a lipophilic sex-steroid. Historically this necessitated the administration in oil, which, in the context of IVF, has traditionally been performed per oral or vaginal application of suppositories or gel, respectively, or by intra-muscular injection.

More recently, progesterone became available as a water-soluble compound for subcutaneous (s.c.) injection [2]. The application of a daily dose of 25 mg s.c. progesterone, equivalent to the physiologic amount produced daily by the ovary in mid-luteal phase, resulted in predecidual changes in 100% of interpretable endometrium samples in a dose finding study [3].

In the year 2013, two large phase III studies (07EU/Prg06 and 07USA/Prg05) on s.c. progesterone were finalized [4, 5]. Both studies were designed and conducted to establish non-inferiority of ongoing pregnancy likelihood in patients undergoing IVF or ICSI and receiving luteal phase support with daily s.c. injections of 25 mg progesterone as compared to vaginally administered progesterone gel [4] or progesterone tablets [5]. In order to demonstrate non-inferiority of a new drug, a margin of non-inferiority for a clinically relevant outcome needs to be pre-specified, and, once a study has been completed, the lower margin of the 95% confidence interval of the absolute difference between treatments to be compared should lie entirely on the positive side of the pre-specified non-inferiority margin. In the context of IVF, where the relevant outcome is binary by nature (e.g. pregnancy or no pregnancy is achieved), relatively large sample sizes are needed to exclude degrees of inferiority which most clinicians and patients would find unacceptable [6]. As a trade-off between the feasibility of new drug development in IVF and the risk of true inferiority, most recent licensing studies in IVF have pre-specified -10% [4,7,8,9] or -8% [10,11] non-inferiority margins for the primary efficacy outcome ongoing pregnancy rate (at expected control group pregnancy rates of typically 30%). Both phase III studies on s.c. progesterone have accordingly established non-inferiority with regard to a -10% margin for ongoing pregnancy rates. However, for the practising clinician, a higher level of confidence into the relative efficacy of a new compound is certainly desirable, which can be achieved by combining study results, thereby yielding more precise and more reliable estimates of true underlying treatment effects. Therefore, a meta-analysis of the two phase III studies on the level of individual patient data (IPD) is presented hereafter.

## Materials and Methods

This IPD meta-analysis combines data from two phase III trials (07EU/Prg06, NCT00827983; 07USA/Prg05, NCT00828191) performed according to GCP standards, resulting in a total sample size of 1483 randomized patients, 1435 of whom underwent embryo transfer and is reported according to PRISMA guidelines. Outcomes of interest are ongoing pregnancy rate, live birth rate and OHSS risk. The protocol for this IPD meta-analysis was not registered.

### Literature search for further studies

**Fig 1:**
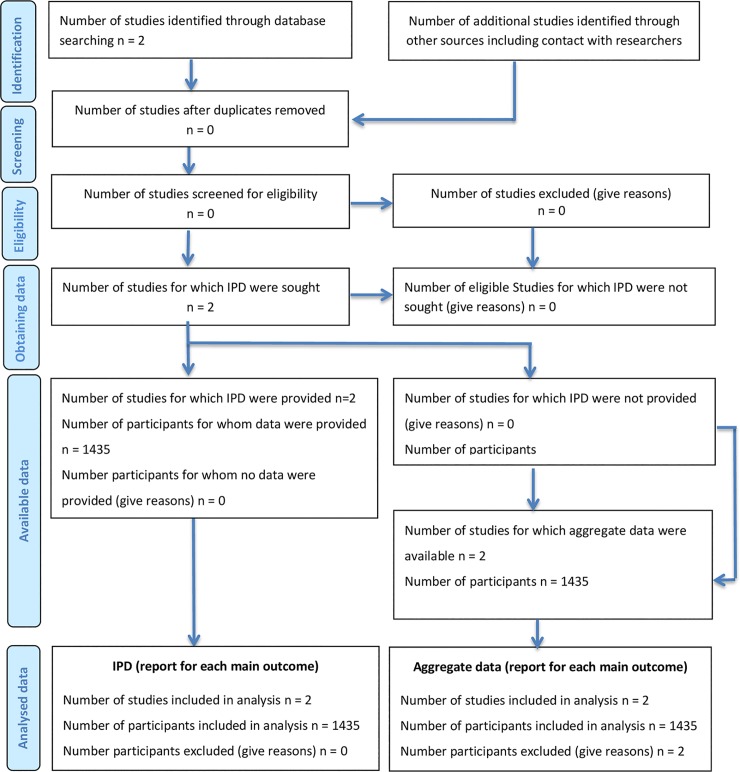
Flow Diagram of literature search and study inclusion according to PRISM guidelines for individual patient data meta-analyses.

Subcutaneous progesterone has only been introduced to the market in 2014 in selected countries making the conduct, data collection analysis and publication of an investigator initiated randomized interventional study virtually impossible within the short time frame, but nevertheless the following databases were searched in January 2016 for published and unpublished RCTs: the Cochrane Central Register of Controlled Trials, MEDLINE, EMBASE, NIH Clinical Trials registry and Google Scholars. Search strategies were, for example: ((Prolutex) OR Lubion) OR Progedex) OR Inprosub) OR subcutaneous progesterone) OR Luteal phase support) D Luteal*[all]) OR in-vitro fertilisation[MeSH Terms] AND Clinical Trial[ptyp])) limited to 2014–2016. Three ongoing trials were identified in the NIH Clinical Trials registry. No further RCT was identified and accordingly, the IPD meta-analysis was limited to the two available phase III trials (Fig 1, S1 PRISMA Checklist).

### Study characteristics

The first phase III trial, “Subcutaneous Progesterone Versus Vaginal Progesterone Gel for Luteal Phase Support in Patients Undergoing In-Vitro Fertilization (IVF)” (07EU/Prg06, NCT00827983), was designed as a prospective, open, randomised, parallel, multicentre, two arm trial. A total of 683 women between 18 and 42 years of age undergoing IVF were randomized on the day of oocyte retrieval after controlled ovarian stimulation in 13 European sites [4]. Embryo transfer was performed in 640 patients and this number therefore constitutes the per-protocol population. Inclusion criteria were: BMI <30kg/m^2^, <3 prior ART cycles (IVF, ICSI and related procedures), baseline (cycle day 2 or 3) FSH <15 IU/L and E_2_ <80 pg/mL, normal uterine cavity as per recent hysterosalpingogram, sonohysterogram or hysteroscopic exam (i.e. no polyp or protruding sub-mucosal fibroid), at least 3 retrieved oocytes and written informed consent. Significant exclusion criteria included cavity-distorting intramural fibroids, stage III or IV endometriosis, hydrosalpinx, history of previous poor response, recurrent miscarriage, adrenal or thyroid disease, and thromboembolic disease or disorder. After randomization, progesterone 25 mg s.c. once a day or progesterone vaginal gel 90 mg once a day were given for at least two weeks to a maximum of 10 weeks depending on pregnancy test result [4].

The second phase III trial entitled “Subcutaneous Progesterone Versus Vaginal Progesterone Tablets for Luteal Phase Support in In Vitro Fertilization (IVF)” (07USA/Prg05; NCT00828191) similarly was as prospective, open, randomized, parallel, multicentre, two-arm trial [5]. Inclusion and exclusion criteria were also highly similar to the prior described study. Included were women between 18 and 42 years who had given written informed consent and met the following criteria: BMI <30 kg/m^2^,<3 prior ART cycles (IVF, ICSI and related procedures), baseline (day 2–3 of cycling) FSH <15 IU/L and E_2_ <80 pg/mL, normal uterine cavity as per recent hysterosalpingogram, sonohysterogram or hysteroscopic exam (i.e. no polyps or protruding submucosal fibroids), and patients must have had at least three retrieved oocytes. Significant exclusion criteria were: intramural uterine fibroids that distort the uterine cavity or polyps >1 cm, stage III or IV endometriosis (no endometriomas), hydrosalpinges, history of past poor response to controlled ovarian hyperstimulation resulting in cancelling ART, the use of thawed/donated oocytes, and history of recurrent pregnancy loss defined as 3 or more spontaneous miscarriages.

On the day of oocyte retrieval, 800 women between 18 and 42 years from eight U.S. sites were randomized and received either progesterone 25 mg s.c. once a day or 100 mg intravaginal twice a day for a maximum of ten weeks depending on presence of pregnancy. Of these, 782 received embryo transfer.

In both studies, the primary outcome of interest was ongoing pregnancy rate, defined as a viable pregnancy 10 weeks after starting Progesterone treatment.

### Data analysis and statistics

Firstly, the presence of statistical heterogeneity between the trials was tested by summarising treatment effects for each trial using risk differences (RD) obtained from the individual studies. Then an I^2^ statistic was applied to the aggregated data from the two trials to describe the percentage of variation across trials caused by heterogeneity as opposed to sampling error.

Next, demographic and treatment parameters were then compared between treatment groups and between patients achieving or not an ongoing pregnancy or live birth in order to identify potential predictors of a positive treatment outcome. Results are presented descriptively, as n, mean, standard deviation for continuous variables or frequencies and percentages for categorical variables, as appropriate. For continuous variables, differences between groups were analysed by means of Mann-Whitney U-test; for non-ordered categorical variables, the groups were compared using Fisher´s exact test.

Then a logistic regression model was fitted, describing the effect of progesterone on pregnancy likelihood adjusted for trial. Trial was included as a fixed effect dummy that allowed the log odds to vary across the trials. This model was further adjusted separately for women’s age and BMI, previous pregnancies leading to a live birth, duration (months) and cause of infertility (female factor, male factor, unexplained and combined), baseline FSH level, endometrial thickness, number of oocytes retrieved, type of treatment (IVF or ICSI), number of embryos transferred and transfer difficulty. These covariates were included in a stepwise logistic regression modelling procedure to determine multiple covariates that, together, had a significant effect on the outcome. Then, effects for predictors of pregnancy, which resulted statistically significant in this model, were inserted in a logistic model while holding the trial and treatment as effects fixed. The odds ratio and 95% confidence intervals were calculated taking into account all patients from the intent-to-treat population who underwent embryo transfer.

The same approach was used for live birth outcome and for ovarian hyperstimulation syndrome development (OHSS). For the latter, risk factors evaluated were women’s age and BMI, presence of polycystic ovaries, presence of allergies, number of oocytes retrieved and type of stimulation protocol (GnRH Agonist and GnRH Antagonist).

All P values were rounded off to three decimals. Statistical significance was defined as a P value was <0.05. All statistical analyses were performed using the software package SAS^®^ system version 9.4.

## Results

Demographics and treatment characteristics were comparable between the two treatment groups as depicted in Table 1.

**Table 1:** Demographics and treatment characteristics in the total population of the two trials (values are mean ± standard deviation or numbers and proportions).

Parameter	Treatment		P value
	Progesterone s.c. (n = 714)	Progesterone vaginal (n = 721)	
Age (years)	33.8 (4.3)	34.0 (4.4)	0.419
BMI (Kg/m^2^)	23.2 (3.2)	23.3 (3.1)	0.387
Previous children, n (%)	218 (30.53)	222 (30.79)	0.954
Duration of infertility (months)	40.4 (31.2)	41.0 (28.4)	0.148
Baseline FSH (IU/ml)	7.0 (2.2)	7.0 (2.1)	0.830
No. of oocytes retrieved	13.3 (8.1)	13.0 (7.5)	0.613
No. of embryos transferred	2.2 (0.7)	2.1 (0.7)	0.422
Endometrial thickness (mm)	11.01 (2.23)	11.15 (2.56)	0.563
Type of treatment, n (%)			0.391
IVF	200 (28.01)	191 (26.49)	
ICSI	421 (58.96)	449 (62.27)	
Both	93 (13.03)	81 (11.23)	
Transfer difficulty, n (%)			0.151
Easy	668 (93.56)	665 (92.23)	
Moderately difficult	42 (5.88)	50 (6.93)	
Extremely difficult	4 (0.56)	2 (0.28)	
Not applicable	0 (0.00)	4 (0.55)	

### Ongoing pregnancy likelihood

In the s.c. progesterone and vaginal progesterone study arms combined, 256/714 and 278/721 patients, respectively, achieved a pregnancy which was ongoing at 10 weeks after the initiation of progesterone treatment. The pooled risk difference for s.c. progesterone versus vaginal progesterone was -0.03 (95% confidence interval -0.08 to 0.02; P = 0.27; Mantel-Haenszel fixed effects model). The I^2^ statistic was 0% (P = 0.99), suggesting no significant heterogeneity between trials.

Comparing patients with or without an ongoing pregnancy in the two trials, it was found that female age (p<0.0001), number of oocytes retrieved (p<0.0001), transfer difficulties (p = 0.037) and endometrial thickness (p = 0.042) differed significantly, or in other words, in cycles that resulted in pregnancy the patients were younger, had more oocytes retrieved, a greater endometrial thickness and a less difficult transfer process. The logistic regression analysis, depicted in Table 2, indicated that older women had a lower odds of pregnancy than younger women (OR = 0.939, 95% CI 0.916 to 0.963; P = < .0001), a finding which remained significant after stepwise selection for confounders (Adj. OR = 0.944, 95% CI 0.919 to 0.969; P = < .0001). The covariates that were included in the adjusted model were trial, age, no. of oocytes retrieved and transfer difficulty. Higher endometrial thickness (OR = 1.056, 95% CI 1.009 to 1.105; P = 0.018) and an increasing number of oocytes retrieved (OR = 1.024, 95% CI 1.009 to 1.039; P = 0.0019), were associated with a higher chance of pregnancy, but this association was no longer significant after adjustment for the above listed confounders. Treatment arm (e.g. the administration of s.c. progesterone versus intra-vaginal progesterone) had no impact on pregnancy likelihood (OR = 0.865, 95% CI 0.694 to 1.077; P = n.s.).

**Table 2:** Predictors of ongoing pregnancy after progesterone treatment. Figures are numbers (percentages) unless stated otherwise.

	Pregnant^1^		Odds ratio (95% CI)	
Parameters	Yes	No	Crude^2^	Adjusted^3^
Randomised treatment:				
Progesterone s.c. vs Progesterone vaginal	256/534 (47.94)	458/901 (50.83)	0.885 (0.713 to 1.098)	0.865 (0.694 to 1.077)
Progesterone vaginal	278/534 (52.06)	443/901 (49.17)	1	1
Median (IQR) age of woman (yrs)	33.00 (30.00–36.00)	34.00 (31.00–38.00)	0.939 (0.916 to 0.963)^4a^	0.944 (0.919 to 0.969)^5^
Median (IQR) BMI of woman	22.94 (21.03 to 25.25)	22.74 (20.64 to 25.54)	1.001 (0.966 to 1.036)	-
Median (IQR) duration of infertility (months)	34.00 (20.00 to 48.00)	36.00 (22.00 to 51.00)	1.000 (0.997 to 1.004)	-
Type of treatment				-
IVF vs Both	131/534 (24.53)	260/901 (28.86)	0.843 (0.579 to 1.227)	
ICSI vs Both	331/534 (61.99)	539/901 (59.82)	1.066 (0.756 to 1.502)	
Both	72/534 (13.48)	102/901 (11.32)	1	
Primary cause of infertility				-
Female vs Unexplained	157/534 (29.40)	248/901 (27.52)	1.172 (0.823 to 1.668)	
Male vs Unexplained	202/534 (37.83)	359/901 (39.84)	1.160 (0.827 to 1.628)	
Combined vs Unexplained	104/534 (19.48)	157/901 (17.43)	1.364 (0.930 to 2.002)	
Unexplained	71/534 (13.30)	137/901 (15.21)	1	
Median (IQR) endometrial thickness (mm)	11.00 (9.80 to 12.30)	10.80 (9.30 to 12.00)	1.056 (1.009 to 1.105)^4b^	-
Previous children				-
Yes	162/534 (30.34)	278/901 (30.85)	1	
No vs Yes	372/534 (69.66)	623/901 (69.15)	0.987 (0.781 to 1.249)	
Median (IQR) baseline FSH level (IU/l)	6.70 (5.60 to 8.04)	6.81 (5.60 to 8.10)	0.967 (0.920 to 1.018)	-
Median (IQR) No. of oocytes retrieved	13.00 (9.00 to18.00)	11.00 (7.00–16.00)	1.024 (1.009 to 1.039)^4c^	1.015 (0.999 to 1.031)
Median (IQR) No. of embryos transferred	2.00	2.00 (2.00 to 3.00)	0.878 (0.755 to 1.022)	-
Transfer difficulty				
Easy vs Moderately difficult	509/533 (95.50)	824/898 (91.76)	1.704 (1.045 to 2.781)	1.750 (1.070 to 2.863)
Moderately difficult	23/533 (4.32)	69/898 (7.68)	1	1
Extremely difficult vs Moderatey difficult	1/533 (0.19)	5/898 (0.56)	0.572 (0.063 to 5.216)	0.514 (0.057 to 4.679)

^1^Median (IQR = interquartile range) presented for skewed data. Denominator differs because of missing values for some characteristics

^2^Estimated from separate logistic models adjusted for Progesterone s.c. vs Progesterone vaginal and trial

^3^Adjusted for Progesterone s.c. vs Progesterone vaginal, trial, age, no. of oocytes retrieved and transfer difficulty, where treatment and trial were considered as fixed effects and the other variables were resulted predictors of pregnancy from a stepwise logistic regression

^4a^ P value < .0001

^4b^ P value = 0.018

^4c^ P value = 0.0019

^5^P value < .0001

### Live birth likelihood

In the s.c. progesterone and vaginal progesterone study arms combined, 252/714 and 271/721 patients, respectively, achieved a live birth. The pooled risk difference for s.c. progesterone versus vaginal progesterone was -0.02 (95% confidence interval -0.07 to 0.03; P = 0.34; Mantel-Haenszel fixed effects model). The I^2^ statistic was 0% (P = 0.87), suggesting no significant heterogeneity between trials.

Similarly to the analysis on ongoing pregnancy rate, female age (p<0.0001), number of oocytes retrieved (p<0.0001) and endometrial thickness (P = 0.05) were significantly different between patients with and without live birth. Table 3 depicts the result of the logistic regression analysis in analogy to the paragraph on ongoing pregnancy rate. The odds of having a live birth were less for older women (OR = 0.940, 95% CI 0.917 to 0.964; P = < .0001). The result for age did not change substantially when the analysis was also adjusted for no. of oocytes retrieved and transfer difficulty (Adj. OR 0.945, 95% CI 0.920 to 0.970; P = < .0001). Live birth rate improved with increased endometrial thickness (1.054, 95% CI 1.007 to 1.103; P = 0.0225) and an increase in the number of oocytes (OR = 1.021, 95% CI 1.006 to 1.037; P = 0.0062). These differences did not remain statistically significant after adjusting for age, number of oocytes retrieved and transfer difficulty (Adj. OR = 1.012, 95% CI 0.996 to 1.028; P = n.s.). Treatment arm (e.g. the administration of s.c. progesterone versus intra-vaginal progesterone) had no impact on live birth likelihood (OR = 0.889, 95% CI 0.714 to 1.106; P = n.s.).

**Table 3:** Predictors of live birth after progesterone treatment. Figures are numbers (percentages) unless stated otherwise.

	Live birth^1^		Odds ratio (95% CI)	
Parameters	Yes	No	Crude^2^	Adjusted^3^
Randomised treatment:				
Progesterone s.c. vs Progesterone vaginal	252/523 (48.18)	462/912 (50.66)	0.900 (0.725 to 1.118)	0.889 (0.714 to 1.106)
Progesterone vaginal	271/523 (51.82)	450/912 (49.34)	1	1
Median (IQR) age of woman (yrs)	33.00 (30.00–36.00)	34.00 (31.00–38.00)	0.940 (0.917 to 0.964) ^4a^	0.945 (0.920 to 0.970) ^5^
Median (IQR) BMI of woman	22.85 (21.00 to 25.25)	22.79 (20.66 to 25.52)	0.996 (0.962 to 1.032)	-
Median (IQR) duration of infertility (months)	34.00 (20.00 to 48.00)	36.00 (22.00 to 51.00)	1.000 (0.997 to 1.004)	-
Type of treatment				-
IVF vs Both	130/523 (24.86)	261/912 (28.62)	0.851 (0.584 to 1.240)	
ICSI vs Both	322/523 (61.57)	548/912 (60.09)	1.040 (0.737 to 1.467)	
Both	71/523 (13.58)	103/912 (11.29)	1	
Primary cause of infertility				-
Female vs Unexplained	155/523 (29.64)	250/912 (27.41)	1.225 (0.858 to 1.748)	
Male vs Unexplained	198/523 (37.86)	363/912 (39.80)	1.199 (0.852 to 1.687)	
Combined vs Unexplained	102/523 (19.50)	159/912 (17.43)	1.407 (0.956 to 2.070)	
Unexplained	68/523 (13.00)	140/912 (15.35)	1	
Median (IQR) endometrial thickness (mm)	11.00 (9.80 to 12.30)	10.80 (9.30 to 12.00)	1.054 (1.007 to 1.103) ^4b^	-
Previous children				-
Yes	159/523 (30.40)	281/912 (30.81)	1	
No vs Yes	364/523 (69.60)	631/912 (69.19)	0.983 (0.776 to 1.244)	
Median (IQR) baseline FSH level (IU/l)	6.70 (5.60 to 8.08)	6.81 (5.60 to 8.10)	0.970 (0.922 to 1.021)	-
Median (IQR) No. of oocytes retrieved	13.00 (9.00 to18.00)	11.00 (7.00–16.00)	1.021 (1.006 to 1.037)^4c^	1.012 (0.996 to 1.028)
Median (IQR) No. of embryos transferred	2.00	2.00 (2.00 to 3.00)	0.888 (0.762 to 1.034)	**-**
Transfer difficulty				**-**
Easy vs Moderately difficult	498/522 (95.40)	835/909 (91.86)	1.645 (1.008 to 2.684)	
Moderately difficult	23/522 (4.41)	69/909 (7.59)	1	
Extremely difficult vs Moderately difficult	1/522 (0.19)	5/909 (0.55)	0.570 (0.063 to 5.199)	

^1^Median (IQR = interquartile range) presented for skewed data. Denominator differs because of missing values for some characteristics

^2^Estimated from separate logistic models adjusted for Progesterone s.c. vs Progesterone vaginal and trial

^3^Adjusted for Progesterone s.c. vs Progesterone vaginal, trial, age, no. of oocytes retrieved and transfer difficulty, where treatment and trial were considered as fixed effects and the other variables were resulted predictors of live birth from a stepwise logistic regression

^4a^ P value < .0001

^4b^ P value = 0.0225

^4c^ P value = 0.0062

^5^P value < .0001

### OHSS risk

The incidence of OHSS of different severity was comparable between groups as depicted in Table 4. In the 07EU/Prg06 trial 5/322 and 8/331 patients in the s.c. progesterone and vaginal progesterone study arm, respectively, were reported as having OHSS of any grade. In the 07USA/Prg05 trial OHSS was reported in 22/392 and in 18/390 patients in the s.c. progesterone and vaginal progesterone study arm, respectively. The pooled odds ratio for s.c. progesterone versus vaginal progesterone was 1.04 (95% confidence interval 0.60 to 1.81; P = 0.88; Mantel-Haenszel fixed effects model). The I^2^ statistic was 0% (P = 0.32), indicating no heterogeneity between trials.

**Table 4:** Incidence of OHSS by grade in the two trials and combined.

		Progesterone s.c	Progesterone vaginal	Total	P-value
07EU/Prg06	Number of patients	322	331	653	0.578
	Total number of events	5 (1.55%)	8 (2.42%)	13 (1.99%)	
	Mild	1 (0,31%)	2 (0,60%)	3 (0,46%)	
	Moderate	1 (0,31%)	5 (1,51%)	6 (0,92%)	
	Severe	3 (0,93%)	1 (0,30%)	4 (0,61%)	
07USA/Prg05	Number of patients	392	390	782	0.627
	Total number of events	22 (5.61%)	18 (4.62%)	40 (5.12%)	
	Mild	11 (2,81%)	8 (2,05%)	19 (2,43%)	
	Moderate	9 (2,30%)	7 (1,79%)	16 (2,05%)	
	Severe	2 (0,51%)	3 (0,77%)	5 (0,64%)	
Combined	Number of patients	714	721	1435	0.889*
	Total number of events	27 (3.78%)	26 (3.61%)	53 (3.69%)	
	Mild	12 (1.68%)	10 (1.39%)	22 (1.53%)	0.674**
	Moderate	10 (1.40%)	12(1.66%)	22 (1.53%)	0.831***
	Severe	5 (0.70%)	4 (0.55%)	9 (0.63%)	0.752****

*χ^2^ test on the number of OHSS events considering the study as covariate p = 0.880

**χ^2^ test on the number of mild OHSS events considering the study as covariate p = 0.668

***χ^2^ test on the number of moderate OHSS events considering the study as covariate p = 0.674

****χ^2^ test on the number of OHSS events considering the study as covariate p = 0.728

The logistic regression analysis depicted in Table 5 identified young age, PCO and a large number of oocytes retrieved as factors that predispose to OHSS. Compared with younger patients, older patients have a decreased OHSS risk (OR = 0.872, 95% CI 0.820 to 0.928; P value < .0001); the odds of a woman without polycystic ovaries experiencing OHSS were lower than those of a woman with PCO appearance of the ovaries (OR = 0.332, 95% CI 0.160 to 0.691; P value = 0.0032) and OHSS risk increased with an increase in ovarian response to stimulation as measured by the number of oocytes retrieved (OR = 1.088, 95% CI 1.058 to 1.120; P value < .0001). In the adjusted model, only age and no. of oocytes remained as significant predictors of OHSS risk (Adj OR = 0.912, 95% CI 0.854 to 0.974; P = 0.0064 and Adj OR = 1.073, 95% CI 1.041 to 1.107; P < .0001). Treatment arm (e.g. the administration of s.c. progesterone versus intra-vaginal progesterone) had no impact on the OHSS risk (OR = 0.995, 95% CI 0.565 to 1.754; P = n.s.).

**Table 5:** Predictors of onset of OHSS after progesterone treatment. Figures are numbers (percentages) unless stated otherwise.

	OHSS^1^		Odds ratio (95% CI)	
Parameters	Yes	No	Crude^2^	Adjusted^3^
Randomised treatment:				
Progesterone s.c. vs Progesterone vaginal	27/53 (50.94)	687/1382 (49.71)	1.043 (0.602 to 1.810)	0.995 (0.565 to 1.754)
Progesterone vaginal	26/53 (49.06)	695/1382 (50.29)	1	1
Median (IQR) age of woman (yrs)	32.00 (29.00–34.00)	34.00 (31.00–37.00)	0.872 (0.820 to 0.928) ^4a^	0.912 (0.854 to 0.974) ^5a^
Median (IQR) BMI of woman	22.30 (20.99 to 25.00)	22.86 (20.76 to 25.40)	0.975 (0.890 to 1.067)	-
Polycystic ovaries				
Yes	10/53 (18.87)	86/1382 (6.22)	1	-
No vs Yes	43/53 (81.13)	1296/1382 (93.78)	0.332 (0.160 to 0.691)^4b^	-
Allergies				
Yes	18/53 (33.96)	287/1382 (20.77)	1	
No vs Yes	35/53 (66.04)	1095/1382 (79.23)	0.739 (0.393 to 1.387)	
Medication type				-
GnRH-Agonist vs Both	43/53 (81.13)	1049/1381 (75.96)	0.649 (0.082 to 5.140)	
GnRH-Antagonist vs Both	9/53 (16.98)	320/1381 (23.17)	0.464 (0.054 to 3.991)	
Both	1/53 (1.89)	12/1381 (0.87)	1	
Median (IQR) No. of oocytes retrieved	18.00 (12.00 to 27.00)	11.00 (7.00–17.00)	1.088 (1.058 to 1.120)^1^^4a^	1.073 (1.041 to 1.107)^5b^

^1^Median (IQR = interquartile range) presented for skewed data. Denominator differs because of missing values for some characteristics

^2^ Estimated from separate logistic models adjusted for Progesterone s.c. vs Progesterone vaginal and trial

^3^ Adjusted for Progesterone s.c. vs Progesterone vaginal, trial, age and no. of oocytes retrieved, where treatment and trial were considered as fixed effects and the other variables were resulted predictors of OHSS from a stepwise logistic regression

^4a^ P value < .0001

^4b^ P value = 0.0032

^5a^ P value = 0.0064

^5b^ P value < .0001

## Discussion

The recent market introduction of subcutaneous progesterone for luteal phase support in IVF broadens the spectrum of treatment options for women undergoing IVF treatment, especially for those women who do not tolerate or who dislike vaginal formulations. It is of great importance though that equal efficacy and safety of a new formulation is established in studies of high methodological quality before a new compound is used on a larger scale. Herein it is shown on individual patient data level that differences between s.c. and vaginal progesterone in pregnancy likelihood are small and indistinguishable from chance when combining two large phase III trials of similar methodology conducted in a similar patient population. In contrast to conventional meta-analyses, in which aggregate study level data are synthesized, the present study models patient level data, while still accounting for clustering of patients within studies. Furthermore, the likelihood of a positive treatment outcome or an adverse event could be adjusted according to well known confounders, an aspect which is not possible in conventional meta-analysis.

For the most relevant efficacy parameter live birth rate, only female age (e.g. chronological age) and ovarian response (e.g. biological age) both independently impacted on the likelihood of live birth in the two trials. This observation is consistent with study results reporting that AMH, a proxy for biological age, is an independent predictor of live birth in patients undergoing IVF [12]. Of note, the type of progesterone used for luteal support did not alter the chance of a patient of achieving a live birth. This is in agreement with the results already presented by Van der Linden et al. [1]

For the risk of OHSS, female age and ovarian response were found to be independent significant risk modifiers, while no impact was found for the type of progesterone used. The risk factor polycystic ovaries did not remain in the final model since the number of oocytes retrieved was a stronger risk modifier. It is interesting to see that the use of either GnRH-agonist or GnRH-antagonist protocols was not associated with the risk of OHSS, while a lower risk of OHSS has consistently been found after GnRH-antagonist stimulation in a large number of RCTs [13, 14]. This can be explained by the fact that the protocol choice was left to the study doctor in both phase III trials and patients identified at risk of ovarian hyperresponse and OHSS had most likely not been put on a GnRH-agonist protocol.

Both trials included in this meta-analysis had allowed the inclusion of a rather broad spectrum of patients (e.g. age 18–42) and pre-randomization treatments (e.g. types of gonadotropin and stimulation protocol). This is not necessarily a disadvantage but rather strengthens the external validity of the results from the combined analysis presented herein. Furthermore, both phase III studies were remarkably homogenous in clinical (patient population, doses of the IP tested etc.) as well as in statistical (e.g. effect directions and effect sizes) terms, which established the basis for a sensible pooling of the patient data.

Finally, it is important to note that at the time of writing no further clinical study on the use of s.c. progesterone has been published, though a large number of investigator-initiated trials have been registered in international trial registries. It can be expected that the use of s.c. progesterone will be studied in various clinical scenarios including frozen-thawed embryo replacement cycles, oocyte donation cycles and early pregnancy supplementation.

## Conclusion

The administration of subcutaneous progesterone 25mg per day for luteal phase support is as efficacious and safe as vaginal progesterone gel or capsules.

## Supporting Information

S1 PRISMA Checklist PRISMA checklist. (DOCX)
